# Epipolar Resampling of Cross-Track Pushbroom Satellite Imagery Using the Rigorous Sensor Model

**DOI:** 10.3390/s17010129

**Published:** 2017-01-11

**Authors:** Mojtaba Jannati, Mohammad Javad Valadan Zoej, Mehdi Mokhtarzade

**Affiliations:** Faculty of Geodesy and Geomatics, K. N. Toosi University of Technology, Tehran 19667-15433, Iran; valadanzouj@kntu.ac.ir (M.J.V.Z.); m_mokhtarzade@kntu.ac.ir (M.M.)

**Keywords:** epipolar resampling, pushbroom satellite imagery, rigorous sensor model, cross track imaging

## Abstract

Epipolar resampling aims to eliminate the vertical parallax of stereo images. Due to the dynamic nature of the exterior orientation parameters of linear pushbroom satellite imagery and the complexity of reconstructing the epipolar geometry using rigorous sensor models, so far, no epipolar resampling approach has been proposed based on these models. In this paper for the first time it is shown that the orientation of the instantaneous baseline (IB) of conjugate image points (CIPs) in the linear pushbroom satellite imagery can be modeled with high precision in terms of the rows- and the columns-number of CIPs. Taking advantage of this feature, a novel approach is then presented for epipolar resampling of cross-track linear pushbroom satellite imagery. The proposed method is based on the rigorous sensor model. As the instantaneous position of sensors remains fixed, the digital elevation model of the area of interest is not required in the resampling process. Experimental results obtained from two pairs of SPOT and one pair of RapidEye stereo imagery with different terrain conditions shows that the proposed epipolar resampling approach benefits from a superior accuracy, as the remained vertical parallaxes of all CIPs in the normalized images are close to zero.

## 1. Introduction

The parallax values of the conjugate image points (CIPs) along the baseline of stereo images (horizontal parallax) reveal valuable information about the height of objects; while the vertical parallaxes not only do not convey any useful information, but they can interrupt the stereo-viewing process. The main objective of the epipolar resampling of stereo images is to generate normalized images where the CIPs have no vertical parallaxes and their horizontal parallaxes are linearly proportional with the height of corresponding points in the object space. These are two main applications of epipolar geometry (EG) which provide the possibility of stereo-viewing, automatic image matching, digital elevation model (DEM) generation, and stereo measurements [1,2].

The EG of frame images is well-known and there are well-established procedures for epipolar resampling [3,4] in such a way that given the relative orientation parameters (ROP) of the stereo images and modifying their attitude parameters, vertical parallaxes of CIPs can be eliminated. To this end, the ROP of the stereo imagery are modified so that all conjugate epipolar lines in those stereo images be simultaneously parallelized with the base line of imagery in a common plane [3]. In contrast, linear pushbroom images have a more complicated geometry where each scan line of these images has its own exterior orientation parameters (EOP) [5]. The availability of the GPS/INS information can positively affect the georeferencing of linear pushbroom imagery, however, the dynamic nature of the EOP of these images makes the reconstruction of EG much more complicated [5,6]. The EG of satellite pushbroom imagery was investigated in various studies using the both rigorous and empirical sensor models [6,7,8,9,10,11,12,13,14,15].

Among the rigorous model-based studies, Gupta and Hartley [7], Kim [5], and Habib et al. [8] made efforts to investigate the EG of linear array images by applying the Multiple Projection Centers (MPC) model [9]. In these studies, the trajectory of the sensor was assumed to be straight (i.e., the simplest case). The only difference between the model employed by Kim [5] with the models used in the two other studies is in the way that the sensor’s orientation parameters were modeled. However, these three studies showed two common important results: (1) rigorously derived epipolar lines in scenes captured by linear array scanners are not straight, rather, their shape is hyperbola-like; and (2) each point on a rigorously defined epipolar curve has a specific conjugate epipolar curve (i.e., no conjugate epipolar curves exist). In another study, Lee and Park [10] derived the equation of epipolar lines using the simplified pushbroom sensor model, which is in line with the results of the previous studies. As an important note, no epipolar resampling method has been proposed for linear pushbroom scenes based on the rigorous sensor models till now.

Among the empirical model-based studies, Morgan et al. [11] used the Parallel Projection (PP) model for epipolar resampling of linear pushbroom imagery, while Oh et al. [12], Wang et al. [13], and Koh and Yang [14] employed the Rational Function Model (RFM). In the former study, for a better compliance of the imaging geometry with the PP model, the perspective projection of the cross-track imaging component of original images should firstly be transformed to the parallel one [15]. Such a transformation assumes a flat terrain or a DEM and also assumes a knowledge of the scanner roll angle [11,16,17,18]. Thereafter, transformation parameters from the original to the normalized images can be estimated using a PP model based on a set of CIPs. The three latter studies are based on two features of epipolar curves, namely straightness approximation and local conjugacy [13,19]. Oh et al. [12] determined the direction of approximately conjugate epipolar lines (ACEL) in the stereo imagery space. Because the sampling distance is supposed to be fixed in the image space, the horizontal parallaxes will be linearly proportional to the ground heights, but there is no guarantee the ground axes will be orthogonal [14]. In contrast, Wang et al. [13] determined the direction of ACEL on a virtual horizontal plane in the object space. Because the sampling distance is supposed to be fixed in the object space, the ground axes will be orthogonal, but the horizontal parallaxes may not be proportional to the ground heights [14]. Koh and Yang [14] determined the direction of ACEL in the image space and supposed the sampling distance to be fixed in the ground space. Therefore, this model benefits from the advantages of the two previous models.

In comparison with the Morgan et al. [11] model, the main advantage of RFM-based models is that there is no need to any CIPs to use these models. However, the lack of any physical or geometrical interpretation of the rational function coefficients (RPCs) hinders theoretical investigation of the nature of the EG. Besides, directly determined RPCs are subject to biases in the scanner’s IOP or EOP [20,21,22] and many ground control points (GCPs) are required to indirectly determine these parameters [23], which therefore limits the use of rational functions. In contrast, the PP parameters have a geometrical interpretation, and therefore, this model can explain the EG of linear pushbroom imagery more effectively. However, the PP model does not completely comply with the imaging geometry of linear pushbroom imagery, and only rigorous models describe the scene formation as it actually happens. Therefore, these models are the most accurate ones [8,24] and have been adopted in a variety of applications [25,26,27,28]. Consequently, it is theoretically and practically significant to develop an approach for epipolar resampling of linear pushbroom imagery based on rigorous sensor models.

In this paper, a novel epipolar resampling approach of cross-track linear pushbroom satellite imagery is proposed based on the MPC model. In the proposed method, the vertical parallaxes of CIPs are eliminated by modifying the instantaneous attitude of the stereo imagery. In this way, the required new attitude parameters are computed based on the direction of the IB of each pair of CIPs. Because the instantaneous position of sensors remains unchanged, there is no need for DEM of the ground coverage in the resampling process.

## 2. Theoretical Background

### 2.1. Linear Pushbroom Imaging

In the case of a frame camera, the imaging process is performed by a 2D array of detectors (CCD or CMOS) arranged within its focal plane. In contrast, linear pushbroom scanners have only a long row of detectors within their focal plane; consequently, they capture a 1D (linear) image in a given exposure [29]. A sequence of linear images in the flying direction can be captured by moving the sensor which forms the 2D image frame [29,30]. Therefore, every 1-D image is associated with the specific positional and orientation parameters of the sensor during the time of exposure. Then, each 1-D image will have a distinct set of EOP. This different imaging geometry leads to different mathematical modeling of linear pushbroom imagery. Toward this end, a wide variety of mathematical models have been developed which generally fall into two main rigorous and empirical models [31,32]. Reconstruction of the geometry of an image at the time of imaging is the backbone of rigorous models. Two well-known approaches in this area encompass Orbital Parameters [27,33,34] and Multiple Projection Centers models [6,7,8,9,35]. In the first case, the trajectory of the sensor is modeled based on Keplerian orbital parameters and time-dependent (temporal) polynomials are used to estimate attitude parameters. In the second type of rigorous models, both the sensor trajectory and attitude parameters are estimated using temporal polynomials. In contrast to these models, no physical or geometrical interpretation stands behind empirical models and they attempt to estimate unknown parameters of the model by fitting it to the rigorous sensor model or a set of GCPs. Numerous research studies have been carried out on investigation of the empirical models in the last decade [36,37,38,39].

### 2.2. MPC Model

**Figure 1:**
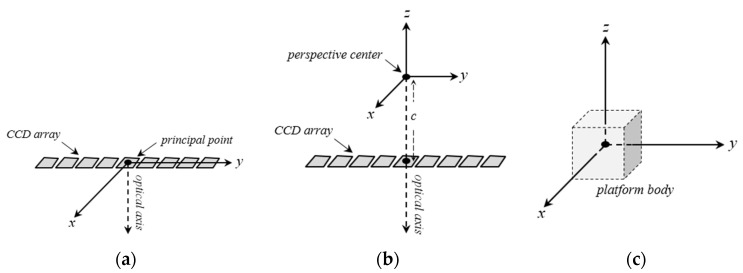
The coordinate systems used in the MPC: (**a**) The IRF; (**b**) The SRF; (**c**) The PRF.

In the MPC model, each scanline of linear pushbroom imagery is supposed as a central perspective image, which has a set of unique EOP. Therefore, each scanline has a distinct Image Reference Frame (IRF), which *x* and *y* axes are directed toward the satellite’s instantaneous velocity vector and the array of sensor’s detectors, respectively. The origin of IRF is the principal point on the image space, which is usually considered at the middle of scanline (Figure 1a). In this model, any arbitrary Ground Reference Frame (GRF) can be used to explain the object space’s coordinates. For convenience in later computations, it is usually adopted according to a map coordinate system (e.g., UTM).

In order to establish the relation between IRF and GRF, two mediator coordinate systems are defined: Sensor Reference Frame (SRF) and Platform Reference Frame (PRF). SRF is a pseudo-3D coordinate system with its origin on the exposure station. Its *x* and *y* axes coincide with the corresponding axes of IRF, and its *z* axis passes through the instantaneous exposure station (Figure 1b). The relationship between IRF and SRF is established based on the IOP of the sensor. PRF is co-origin with the SRF, and its axes are locked to the body of the platform (Figure 1c).

Like in aerial photogrammetry, the sensor is supposed to be locked to the body of the platform in the MPC. Therefore, the axes of SRF and PRF coincide during the acquisition time. Thus, the 3D rotation matrix required to transform from SRF to PRF will be an identity matrix, given by Equation (1):

(1) $$[R_{\text{Attitude}}]=I_{3}=\left[\begin{array}{ccc} 1 & 0 & 0\\ 0 & 1 & 0\\ 0 & 0 & 1 \end{array}\right],$$

where [*R*_Attitude_] is a 3D rotation matrix from the SRF to the PRF.

Transformation from the PRF to the GRF is performed using a 3D rotation matrix through modeling the orientation parameters of the sensor (Equation (2)):

(2) $$\left[R_{\text{Orientation}}]=R_{1}(\omega_{t})\times R_{2}(\phi_{t})\times R_{3}(\kappa_{t}\right),$$

where *ω_t_*, *ϕ_t_*, and *κ_t_* are the orientation angles of the sensor around the *x*, *y* and *z* axes of the GRF, respectively. *R_r_* (for *r* = 1, 2, 3) is a 3D rotation matrix around the *r*-th axis of the GRF. Finally, [*R*_Orientation_] is a 3D rotation matrix from the PRF to the GRF. In practice, the orientation angles *ω_t_*, *ϕ_t_*, and *κ_t_* are modeled as time-dependent polynomials, given by Equation (3):

(3) $$\begin{array}{l} \omega_{t}=\omega_{0}+\omega_{1}t+\omega_{2}t^{2}+\ldots \\ \phi_{t}=\phi_{0}+\phi_{1}t+\phi_{2}t^{2}+\ldots \\ \kappa_{t}=\kappa_{0}+\kappa_{1}t+\kappa_{2}t^{2}+\ldots \end{array},$$

where *t* is the time parameter which can be considered equivalent to the rows-number of the image points. Obviously, the type and number of essential terms included in the polynomials depend on the perturbations that occurred during image formation [29].

Given the known relation between the SRF, the PRF and the GRF, the overall structure of the co-linearity equation can be expressed using Equation (4):

(4) $${\left(\begin{array}{c} X-X_{S}\\ Y-Y_{S}\\ Z-Z_{S} \end{array}\right)}_{\text{GRF}}=\lambda [R_{\text{Orientation}}]\times [R_{\text{Attitude}}]\times {\left(\begin{array}{c} x=0\\ y\\ -c \end{array}\right)}_{\text{SRF}},$$

where (*x* = 0, *y*) is the coordinate of points in the SRF, *c* is the principal distance of the sensor, *λ* is the scale factor, (*X*, *Y*, *Z*) is the object space coordinate in the GRF, and finally, (*X_S_*, *Y_S_*, *Z_S_*) is the instantaneous exposure station in the GRF which is modeled by the time-dependent polynomials [29] given by Equation (5):

(5) $$\vec{S_{t}}=\left(\begin{array}{c} X_{S}\\ Y_{S}\\ Z_{S} \end{array}\right)=\left(\begin{array}{c} X_{0}\\ Y_{0}\\ Z_{0} \end{array}\right)+\left(\begin{array}{c} X_{1}\\ Y_{1}\\ Z_{1} \end{array}\right)\times t+\left(\begin{array}{c} X_{2}\\ Y_{2}\\ Z_{2} \end{array}\right)\times t^{2},$$

where *X_r_*, *Y_r_* and *Z_r_* (for *r* = 0, 1, 2) are the unknown coefficients of the polynomials, and *t* is the time parameter which can be considered equivalent to the rows-number of the image points.

### 2.3. The EG of Linear Pushbroom Images

**Figure 2:**
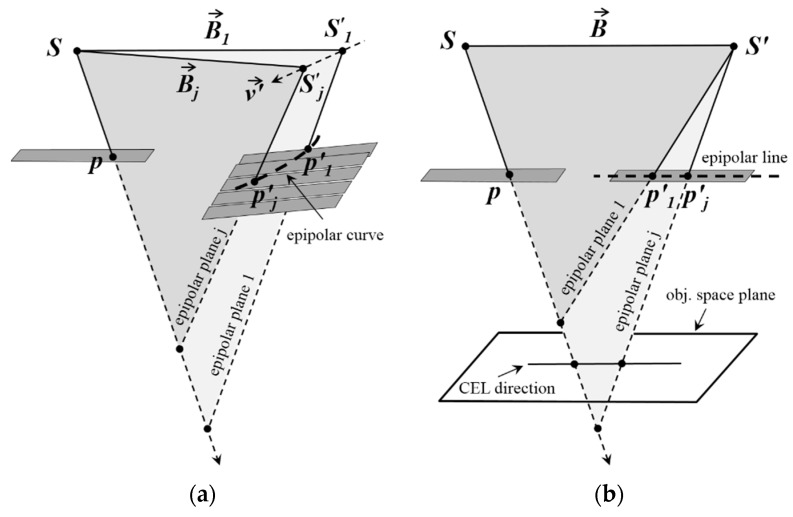
The EG of cross-track linear pushbroom imagery: (**a**) General case; (**b**) Ideal case.

The main difference between frame-type cameras and linear pushbroom sensors is that each scanline of linear imagery has a distinct perspective center. As a result of this characteristic, there will be several epipolar planes on the second scene for a given point p in the first scene [1], and hyperbola-shape epipolar curves (instead of epipolar lines) of linear imagery is due to the non-coplanarity of these planes [1,8] (Figure 2a). For cross-track stereo coverage, the necessary and sufficient condition for coplanarity of all epipolar planes and formation of straight epipolar lines is that the scanlines containing CIPs to be parallel with their IB in a common plane (Figure 2b).

**Figure 3:**
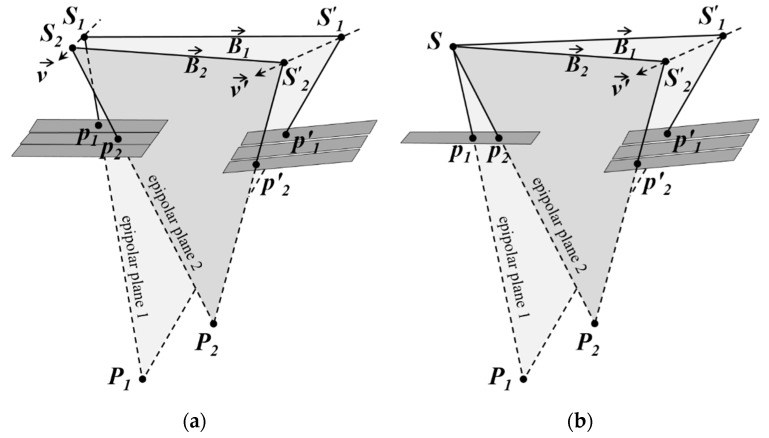
Variations in the direction of the IB of CIPs in the linear stereo scenes: (**a**) Temporal variations; (**b**) Spatial variations.

With a little attention to Figure 2b it can be easily understood that for a given pair of CIPs, the aforementioned condition can be reconstructed by modifying the attitude parameters of scanlines containing the CIPs. In this way, the epipolar curves of these CIPs will convert to straight Conjugate Epipolar Lines (CEL); and the plane containing all possible epipolar planes of these points will intersect any horizontal plane of the object space in a straight line parallel with their IB (Figure 2b). Hereafter, this direction is called the CEL’s direction. Due to the dynamism of two sensors during the capture of stereo scenes, by changing the rows-number of image points, the direction of IB of CIPs will change, as well as their CEL’s direction (Figure 3a). Moreover, given two distinct points such as *p*_1_ and *p*_2_ in the *i*-th scanline of the first scene, their conjugate points will not necessarily be in a unique scanline of the second scene (Figure 3b). Thus, by changing the columns-number of image points, the direction of IB of CIPs and their CEL’s direction will change too. From now on, these two changes in the CEL’s direction will be called the temporal and spatial variations (due to the change of rows- and columns-number of CIPs, respectively) of the IB of CIPs, respectively.

The necessary and sufficient condition for eliminating the vertical parallaxes of all CIPs throughout the linear cross-track stereo imagery is that the epipolar lines of all image points be parallel with each other, and aligned along the scene rows. Therefore, when the attitude parameters of stereo scenes are modified, the vertical parallax of all CIPs can be eliminated indiscriminately by applying a complementary rotation. Due to the temporal and spatial variations of the direction of IB of the CIPs, the required rotation angle will obviously be a function of both the rows- and the columns-number of CIPs.

**Figure 4:**
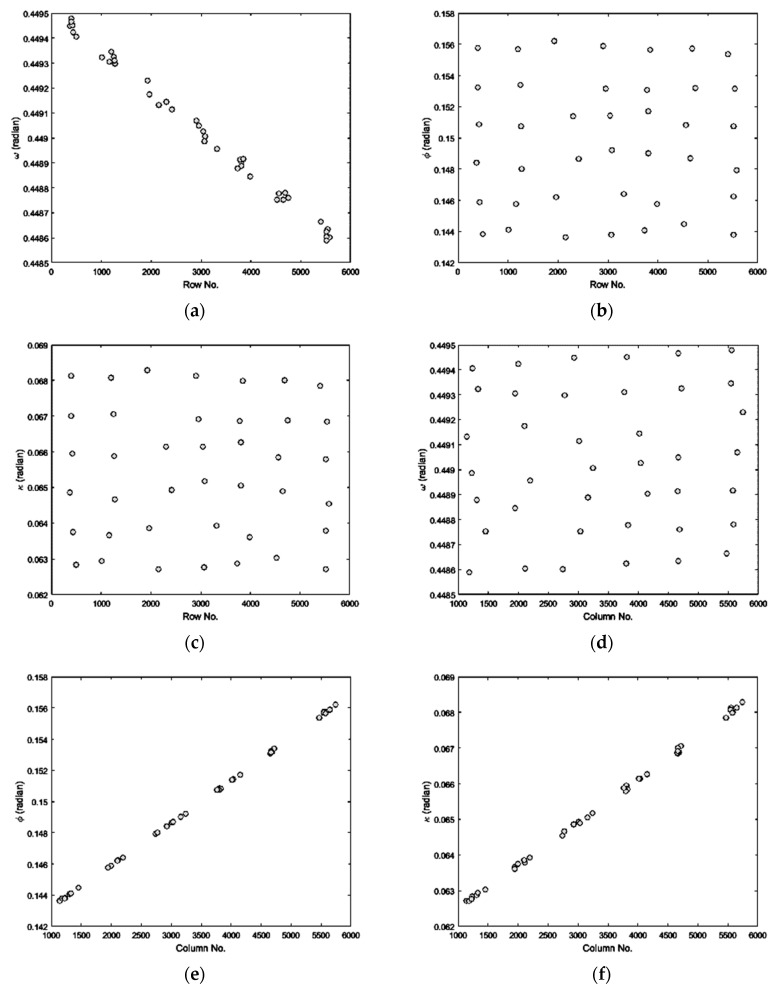
Scatter plot of the direction of IBs with respect to the rows- and columns-number of CIPs: (**a**) *ω* vs. the rows-number; (**b**) *ϕ* vs. the rows-number; (**c**) *κ* vs. the rows-number; (**d**) *ω* vs. the columns-number; (**e**) *ϕ* vs. the columns-number; (**f**) *κ* vs. the columns-number.

As satellite scenes are usually acquired in a very short time and they benefit from a fairly stable attitude [8,29], the temporal and spatial variations of the IB’s direction of CIPs can be modeled using some polynomials in the both rows- and columns-number of CIPs. To validate this theoretical consideration, the IB’s direction of some pairs of CIPs from a real dataset (the left scene of the Isfahan dataset, which is introduced in Section 4) were calculated in terms of three orientation parameters roll (*ω*), pitch (*ϕ*), and yaw (*κ*) of the sensor. The scatter plots of computed parameters are illustrated in Figure 4, with respect to the rows- and the columns-number of CIPs.

**Figure 5:**
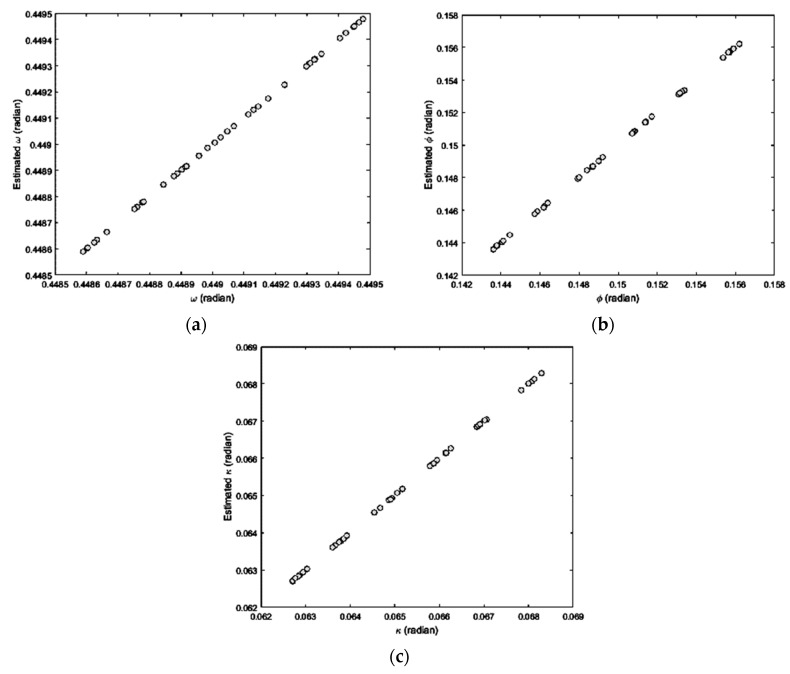
Scatter plot of computed and estimated attitude parameters of the IB of CIPs: (**a**) Attitude parameter *ω*; (**b**) Attitude parameter *ϕ*; (**c**) Attitude parameter *κ*.

According to Figure 4, no one of the attitude parameters *ω*, *ϕ*, and *κ* can exactly be modeled in terms of only one of the rows- or the columns-number of CIPs. Thereafter, some polynomials in the rows- and the columns-number of CIPs were used to estimate the computed attitude parameters. The scatter plot of computed and estimated attitude parameters of the IB of CIPs is shown in Figure 5, which illustrates the quality of estimation.

In order to a quantitative assessment, the obtained coefficients of the polynomials fitted to each of the attitude parameters are provided in Table 1, along with their significance values (*p*-value), standard errors (std-err), coefficients of determination (Pseudo-*R*^2^), and the root mean squares of residuals from the fitted straight lines (${\hat{\sigma }}_{0}$); which confirm the high accuracy of the modeling.

According to Table 1, since the IB’s direction of CIPs has a systematic behavior, the complementary rotation angle required for parallelizing the epipolar lines of CIPs can be modeled using some polynomials in the both rows- and columns-number of CIPs, as well as the new attitude parameters required to parallelizing the scanlines containing the CIPs with their IBs.

## 3. Proposed Method

Suppose two conjugate image points *p* and *p*′, which are respectively in the *i*-th and the *j*-th scanlines of the first and the second imagery. The proposed epipolar resampling method for cross-track linear imagery is constructed in three steps:

- parallelizing scanlines containing the CIPs with their IBs to produce straight epipolar lines,
- parallelizing the epipolar lines of all CIPs with each other to eliminate the vertical parallax of CIPs, and,
- correcting the scale of the normalized scenes.

These steps are explained in more details in the following subsections.

### 3.1. Producing Straight Epipolar Lines

In the first step, the *y* axis of scanlines containing the CIPs should be parallelized with their IB in a common plane, by modifying the attitude parameters of the sensor (Figure 2b). In this way, the direction of IB of CIPs should be computed firstly. Therefore, given the EOP of the stereo imagery, the exposure stations of the *i*-th scan line of the first scene and the *j*-th scan line of the second scene, are calculated using Equation (5). Then, the IB of points *p* and *p*′ is computed using Equation (6):

(6) $${\left(\begin{array}{c} B_{X}\\ B_{Y}\\ B_{Z} \end{array}\right)}_{\text{GRF}}={\vec{B}}_{\text{GRF}}={\vec{S^{\prime }}}_{j}-{\vec{S}}_{i},$$

where *S_i_* and *S*′_*j*_ are the exposure stations of the *i*-th scan line of the first scene and the *j*-th scan line of the second scene, respectively; and *B*_GRF_ is the IB of conjugate points *p* and *p*′ in the GRF.

Since the calculated IB is in the GRF and the new attitude parameters for parallelizing the SRF’s *y* axis of scanlines are required in the PRF, the vector *B*_GRF_ should firstly be transformed to the PRF of each scanline, see Equation (7):

(7) $${\left(\begin{array}{c} B_{X}^{i}\\ B_{Y}^{i}\\ B_{Z}^{i} \end{array}\right)}_{\text{PRF}}={\vec{B}}_{\text{PRF}}^{i}={\left[R_{\text{Orientation}}\right]}_{i}^{T}\times {\vec{B}}_{\text{GRF}},$$

where [*R*_Orientation_]_*i*_ is the rotation matrix [*R*_Orientation_] for the EOP of *i*-th scanline of the first image, *T* is the transpose operator, ${\vec{B}}_{\text{PRF}}^{i}$ is the baseline of the points *p* and *p*′ in the PRF of the *i*-th scanline of the first image, and $B_{X}^{i}$, $B_{Y}^{i}$ and $B_{Z}^{i}$ are the components of the vector ${\vec{B}}_{\text{PRF}}^{i}$ along the *x*, *y* and *z* axes of the PRF, respectively.

Given the ${\vec{B}}_{\text{PRF}}^{i}$, the required attitude parameters for parallelizing the SRF’s *y* axis of the *i*-th scanline of the first image with the IB of CIPs can be calculated using Equations (8) and (9):

(8) $$\omega_{n}^{i}={\text{tan}}^{-1}\left(\frac{B_{Z}^{i}}{B_{Y}^{i}}\right),$$

(9) $$\kappa_{n}^{i}={\text{tan}}^{-1}\left(\frac{B_{X}^{i}}{\sqrt{{B_{Y}^{i}}^{2}+{B_{Z}^{i}}^{2}}}\right),$$

where $\omega_{n}^{i}$ and $\kappa_{n}^{i}$ are the new attitude parameters around the PRF’s *x* and *z* axes of the *i*-th scan line of the first image, respectively.

Moreover, for coplanarity of the *i*-th scanline of the first image and the *j*-th scanline of the second image, it is sufficient that the new attitude parameters around their PRF’s *y* axis be equal. In order to simultaneously account for the off-nadir viewing effect of the sensor, it is recommended that this parameter be considered equal to zero, as indicated by Equation (10):

(10) $$\phi_{n}^{i}=0,$$

where $\phi_{n}^{i}$ is the new attitude parameter around the PRF’s *y* axis of the *i*-th scanline of the first image.

Given the new attitude parameters, the required rotation matrix for parallelizing the SRF’s *y* axis of the *i*-th scanline of the first image with the IB of the conjugate points *p* and *p*′ (i.e., ${\left[R_{\text{Attitud}}^{n}\right]}_{i}$) will be as Equation (11):

(11) $${\left[R_{\text{Attitud}}^{n}\right]}_{i}=R_{1}(\omega_{n}^{i})\times R_{2}(\phi_{n}^{i})\times R_{3}(\kappa_{n}^{i}),$$

In practice, two parameters $\omega_{n}^{i}$ and $\kappa_{n}^{i}$ should firstly be calculated for a set of CIPs, and by fitting a polynomial with a proper order in both the rows- and the columns-number of CIPs then be used in the structure of rotation matrix ${\left[R_{\text{Attitud}}^{n}\right]}_{i}$, Equation (12):

(12) $$\begin{array}{l} \omega^{n}(i,l)=\omega_{0}^{n}+\omega_{1}^{n}\times i+\omega_{2}^{n}\times l+\ldots \\ \kappa^{n}(i,l)=\kappa_{0}^{n}+\kappa_{1}^{n}\times i+\kappa_{2}^{n}\times l+\ldots \end{array},$$

where *i* and *l* are the rows- and the columns-number of CIPs, respectively. $\omega_{r}^{n}$ and $\kappa_{r}^{n}$ (for *r* = 0, 1, 2) are the unknown coefficients of polynomials.

Given the second image’s EOP, the required rotation matrix for parallelizing the SRF’s *y* axis of the *j*-th scanline of the second image with the IB of the conjugate points *p* and *p*′ (i.e., ${\left[R_{\text{Attitud}}^{\prime n}\right]}_{j}$) can similarly be calculated using Equations (8)–(12).

By applying the rotation matrix ${\left[R_{\text{Attitud}}^{n}\right]}_{i}$, the points’ coordinate in the aligned-SRF (i.e., the SRF after than its *y* axis was parallelized with the IB of CIPs) will be as indicated by Equation (13):

(13) $${\left(\begin{array}{c} x_{a}\\ y_{a}\\ -c_{a} \end{array}\right)}_{{\text{SRF}}_{a}}=\frac{\lambda }{\lambda_{a}}{\left[R_{\text{Attitude}}^{n}\right]}^{T}\times {\left[R_{\text{Orientation}}\right]}^{T}\times [R_{\text{Orientation}}]\times [R_{\text{Attitude}}]\times {\left(\begin{array}{c} x=0\\ y\\ -c \end{array}\right)}_{\text{SRF}},$$

where (*x_a_*, *y_a_*, *−c_a_*) is the points’ coordinate in the aligned-SRF (SRF_a_), and *λ_a_* is the scale factor for transferring from GRF to SRF_a_. Since $[R_{\text{Attitude}}]=I_{3}$ and, ${\left[R_{\text{Orientation}}\right]}^{T}\times [R_{\text{Orientation}}]=I_{3}$, Equation (13) can be simplified as Equation (14):

(14) $${\left(\begin{array}{c} x_{a}\\ y_{a}\\ -c_{a} \end{array}\right)}_{{\text{SRF}}_{a}}=k\times {\left[R_{\text{Attitude}}^{n}\right]}^{T}\times {\left(\begin{array}{c} x=0\\ y\\ -c \end{array}\right)}_{\text{SRF}},$$

where $k=\frac{\lambda }{\lambda_{a}}$ is the scale factor for transferring from SRF to SRF_a_.

As previously mentioned, in the SRF_a_ the epipolar curves will become straight epipolar lines which are aligned along the IB of CIPs. 

### 3.2. Eliminating the Vertical Parallax of CIPs

Due to the temporal and spatial variations of the IB’s direction of CIPs, the CEL’s direction throughout the linear stereo imagery will be variable as well. Therefore, in order to the vertical parallaxes of all CIPs be eliminated at ones, it is necessary that all the CELs be parallelized by applying a complementary rotation around the SRF_a_’s *z* axis (Equation (15)):

(15) $${\left(\begin{array}{c} x_{p}\\ y_{p}\\ -c_{p} \end{array}\right)}_{{\text{SRF}}_{p}}=k\times R_{3}(\theta_{n})\times {\left(\begin{array}{c} x_{a}\\ y_{a}\\ -c_{a} \end{array}\right)}_{{\text{SRF}}_{a}}=k\times \left(\begin{array}{c} x_{a}\;\text{cos}(\theta_{n})+y_{a}\;\text{sin}(\theta_{n})\\ y_{a}\;\text{cos}(\theta_{n})-x_{a}\;\text{sin}(\theta_{n})\\ -c_{a} \end{array}\right),$$

where (*x_p_*, *y_p_*, *−c_p_*) is the points’ coordinate in the parallelized-SRF (i.e., the SRF_a_ after that the complementary rotation was applied), $\theta_{n}$ is the complementary rotation angle for parallelizing the CEL throughout the stereo imagery; which in order to account for the temporal and spatial variations of the IB’s direction of CIPs, it is modeled by a polynomial in both the rows- and the columns-number of CIPs (Equation (16)):

(16) $$\theta_{n}=\theta_{0}^{n}+\theta_{1}^{n}\times i+\theta_{2}^{n}\times l+\ldots ,$$

where $\theta_{r}^{n}$ (for *r* = 0, 1, 2, …) are the unknown coefficients of the polynomial.

Given the new attitude parameter of the second imagery, the coordinate of any image point in its parallelized-SRF (SRF_p_) can similarly be calculated using Equations (13)–(16).

The fundamental condition for parallelism of all epipolar lines throughout the stereo imagery is that the vertical parallaxes of all CIPs be equal to a constant value. In order to computing the vertical parallax of CIPs, their *x*-ordinate in the SRF_p_ should firstly be calculated. In this way, by substituting Equation (14) into Equation (15) and dividing the first row of Equation (15) by its third row, the *x*-ordinate of any image point in its SRF_p_ can be computed as Equation (17):

(17) $$x_{p}^{i}=c_{p}\frac{x_{a}\;\text{cos}(\theta_{n})+y_{a}\;\text{sin}(\theta_{n})}{c_{a}},$$

where $x_{p}^{i}$ is the point’s *x*-ordinate in the SRF_p_ of the *i*-th scanline of the first image. 

Since each scanline of linear imagery has a unique SRF, Equation (17) can only be used for relating the SRF of each image point with its SRF_p_. In order to establish the relationship of SRF_p_ of consecutive scanlines, a psudo-2D coordinate system was constructed by arranging the scanlines in a sequential manner (Equation (18)):

(18) $$\begin{array}{l} x_{psud}^{p}=x_{p}^{i}+(i-1)\times D_{CCD}\\ x_{psud}^{\prime p}=x_{p}^{\prime j}+(j-1)\times D_{CCD} \end{array},$$

where *i* and *j* are the rows-number of CIPs in the first and second imagery, respectively; $D_{CCD}$ is the dimension of sensor’s CCD, and finally, $x_{psud}^{p}$ and $x_{psud}^{\prime p}$ are the *x*-ordinate of CIPs in the psudo-2D coordinate system of the first and the second imagery, respectively. By the way, the condition equation of obtaining the parallelized epipolar lines can be written as Equation (19):

(19) $$x_{psud}^{\prime p}-x_{psud}^{p}=d,$$

where *d* is the vertical parallax of CIPs; which should be a constant value.

By substituting Equations (14)–(18) into Equation (19), the final formula to compute the rotation angle $\theta_{n}$ can be written as Equation (20):

(20) $$(j-i)\times D_{CCD}-\left({c^{\prime }}_{p}\frac{{x^{\prime }}_{a}\;\text{cos}({\theta^{\prime }}_{n})+{y^{\prime }}_{a}\;\text{sin}({\theta^{\prime }}_{n})}{{c^{\prime }}_{a}}-c_{p}\frac{x_{a}\;\text{cos}(\theta_{n})+y_{a}\;\text{sin}(\theta_{n})}{c_{a}}\right)-d=0,$$

The above equation is non-linear in terms of the unknowns (i.e., $\theta^{n}$, ${\theta^{\prime }}^{n}$ and *d*), which after differentiation and linearization, can be solved using a set of CIPs. By applying the complementary rotation, CIPs will have no vertical parallax in their SRF_p_. But due to the variations of the IB’s length of CIPs, flying height, and the sensor’s roll angle throughout the stereo imagery, the scale of generated model will be variable. 

### 3.3. Correcting the Scale of the Normalized Scenes

In the third step the model’s scale should be corrected by shifting the CIPs along the y axis of their SRF_p_ (Equation (21)):

(21) $${\left(\begin{array}{c} x_{n}\\ y_{n}\\ -c_{n} \end{array}\right)}_{{\text{SRF}}_{n}}=k\times {\left(\begin{array}{c} x_{p}\\ y_{p}\\ -c_{p} \end{array}\right)}_{{\text{SRF}}_{p}}+\left(\begin{array}{c} 0\\ \Delta y\\ 0 \end{array}\right),$$

where (*x_n_*, *y_n_*, *−c_n_*) the point’s coordinate in the normalized-SRF (SRF_n_), and Δy is the required shift to correct the scale of the point *p* in the first image.

Due to the temporal and spatial variations of the IB of CIPs, the sensor’s roll angle, and flying height, the required shift will obviously be a function of the rows- and the columns-number of CIPs, Equation (22):

(22) $$\Delta y=\Delta y_{0}+\Delta y_{1}\times i+\Delta y_{2}\times l+\Delta y_{3}\times i\times l+\ldots ,$$

where $\Delta y_{r}$ (for *r* = 0, 1, 2) are the unknown coefficients of the polynomial.

In order to computing this parameter, the points’ *y*-ordinate should firstly be calculated in their SRF_p_ (Equation (23)):

(23) $$y_{p}^{i}=c_{p}\frac{x_{a}\;\text{sin}(\theta_{n})+y_{a}\;\text{cos}(\theta_{n})}{c_{a}},$$

where $y_{p}^{i}$ is the points’ *y*-ordinate in the *SRF_p_* of the *i*-th scan line of the first image.

The necessary condition to correct the model’s scale is that after than the CIPs is shifted along the y axis of their SRF_p_, horizontal parallaxes of CIPs and their heights in the object space to be linearly proportional, as indicated by Equation (24):

(24) $$(y_{p}^{\prime j}+\Delta y^{\prime })-(y_{p}^{i}+\Delta y)=aZ+b,$$

where Δy and $\Delta y^{\prime }$ are the required shifts to correct the scales of the first and the second images, respectively, *Z* is the height of CIPs in the GRF, *a* and *b* are the coefficients of the linear relation of horizontal parallaxes and heights. If *b* = 0, and *a* is considered to be equal to the average imaging scale, Equation (24) can be rewritten as:

(25) $$\Delta y^{\prime }-\Delta y=\frac{c}{H_{m}}Z+(y_{p}^{\prime j}-y_{p}^{i}),$$

where *c* is the sensor’s principal distance and *H_m_* is the average flying height.

Finally, by substituting Equations (21)–(24) into Equation (25) and forming a system of equations for a set of CIPs, the required shifts are achieved as a function of both the rows- and the columns-number of CIPs. It should be noted that given the EOP of stereo imagery and the image coordinate of CIPs, the points’ heights in the object space can be calculated via the space intersection of two images. Therefore, no additional GCPs are needed for Equation (25) to be to solved.

Given the new attitude parameters, the complementary rotation angle and the required shift to correct the model’s scale, the final formula to transfer from SRF to SRF_n_ will be as Equation (26):

(26) $${\left(\begin{array}{c} x_{n}\\ y_{n}-\Delta y\\ -c_{n} \end{array}\right)}_{{\text{SRF}}_{n}}=k\times R_{3}(\theta_{n})\times {\left[R_{\text{Attitude}}^{n}\right]}^{T}\times {\left(\begin{array}{c} x=0\\ y\\ -c \end{array}\right)}_{\text{SRF}},$$

where (*x_n_*, *y_n_*, −*c_n_*) is the points’ coordinate in the SRF_n_.

In practice, the invers form of Equation (26) will be used in the epipolar resampling process to transfer image points from normal image space to the original image space and indirect resampling of the linear stereo scenes. In this way, the epipolar resampling procedure can be conducted in a point wise manner as well as the digital rectification process.

## 4. Study Area and Data Used

Three different datasets have been used in this article, including two SPOT-1A stereo imagery sets covering Isfahan and Zanjan provinces, and one RapidEye stereo imagery set covering Fars province, all in Iran. The first and the second datasets cover a foothills rural area with rare urbanized regions. There is a lake at the bottom-right corner of the second dataset, and a little cloud coverage can be seen in this area. The third dataset covers a semi-mountainous foothill area, including two quite big cities almost in the center of the image. A little cloud coverage at the middle-left area of stereo image overlaps can be observed. Cloud percentages, geometric information and the number of available GCPs for each dataset are shown in Table 2, along with their elevation relief.

**Figure 6:**
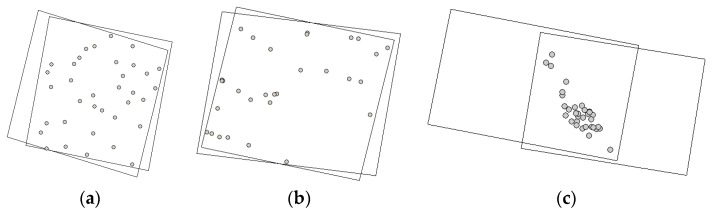
Distribution of GCPs throughout the stereo imagery: (**a**) Isfahan dataset; (**b**) Zanjan dataset; (**c**) Fars dataset.

GCPs of Isfahan and Zanjan datasets were measured using a double-frequency GPS with a sub-meter accuracy, and their corresponding image coordinates were measured with an approximate precision of 0.5 pixel. For the Fars dataset, GCPs were extracted from 1:2000 scale digital topographic maps produced by the National Cartographic Center of Iran with 0.6 m planimetric and 0.5 m altimetric accuracies, respectively. The points are distinct features such as buildings, pool corners, walls, and road junctions. Their corresponding image coordinates were measured with an approximate precision of one pixel. Distribution of GCPs is illustrated in Figure 6. The lack of GCPs in Zanjan and Fars datasets are due to their land coverage.

## 5. Results

According to Section 3, there is no need to any GCPs if the EOP of stereo imagery are available; and the proposed method can be solved using only a few CIPs. In this study, the EOP of imagery were computed using the GCPs. At the space resection stage, in order to optimally structure the rotation matrix [*R*_Orientation_], different structures were investigated in a try and error manner. From the available GCPs for each dataset, 15 points were used at the space resection stage while the remaining points were used as independent check points in the accuracy assessment process. Accuracies obtained from space resection of each image are illustrated in Table 3 in the image space, along with the accuracies obtained from space intersection of the stereo imagery in the object space.

Then, 40 pairs of well distributed CIPs were manually extracted from each stereo imagery set. These points were employed in two different roles. One part of CIPs was used to compute the new attitude parameters, complementary rotation angles, and the required shifts; which is called Control Conjugate Points (CoCP) in this paper. The other part of CIPs, which is called Check Conjugate Points (ChCP), was used to assess the model’s accuracy. In order to evaluate the accuracy of the proposed method as it is impacted by the number of utilized CoCP, several experiments were performed on each dataset. In each experiment, using the parameters which are computed on CoCP, the ChCP’s coordinate were transformed to the SRF_n_. The mean absolute values and the maximum absolute values of the residual vertical parallax (P_V_) of ChCP are provide in Table 4, along with the square root of the estimated variance component from straight-line fitting to horizontal parallaxes (P_H_) of ChCP and their heights in the object space. In this way, the elevation of ChCP were calculated using the space intersection of their image coordinate in the original stereo imagery.

**Figure 7:**
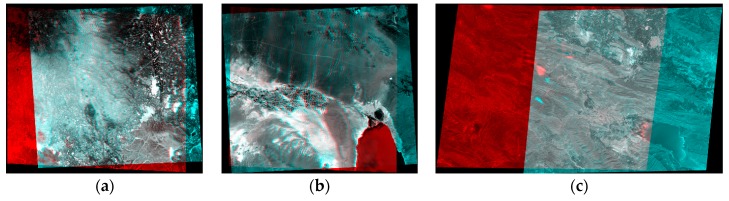
Stereo anaglyphs of the normalized scenes through the Experiments 2, 6, and 10: (**a**) Isfahan dataset; (**b**) Zanjan dataset; (**c**) Fars dataset.

According to Table 4, the proposed method has a high ability to eliminate the vertical parallaxes of CIPs even if a few CoCP are used, so by increasing the number of CoCP from 10 to 16 (i.e., Experiments 3, 4, 7, 8, 11 and 12), no significant improvement is seen in the results. Finally, the produced normalized scenes through the Experiments 2, 6 and 10 were overlayed to generate the corresponding stereo anaglyphs (Figure 7).

## 6. Discussion

**Figure 8:**
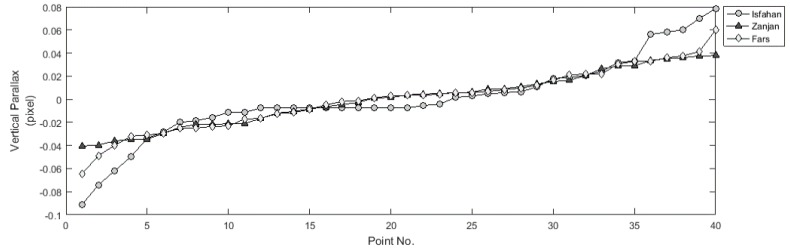
Vertical parallax of CIPs on the normalized scenes in Experiments 2, 6, and 10.

In order to assess the accuracy of the proposed method two factors were examined: (1) the ability to eliminate the vertical parallaxes of CIPs; and (2) to provide a linear relationship between the horizontal parallaxes of CIPs and their heights in the object space. Before applying the proposed epipolar resampling method, the mean values of vertical parallax of CIPs for Isfahan, Zanjan, and Fars datasets were 174, 184.4, and 106.7 pixels, respectively. According to Table 4, in all experiments the maximum absolute values of the residual vertical parallax of normalized scenes are close to zero. Therefore, the proposed method has a high ability to eliminate the vertical parallaxes of CIPs. The residual vertical parallax of CIPs in Experiments 2, 6, and 10 are illustrated in Figure 8, where the horizontal axis shows the points’ number ordered by increasing the residual parallax, while the vertical axis shows the value of vertical parallax.

**Figure 9:**
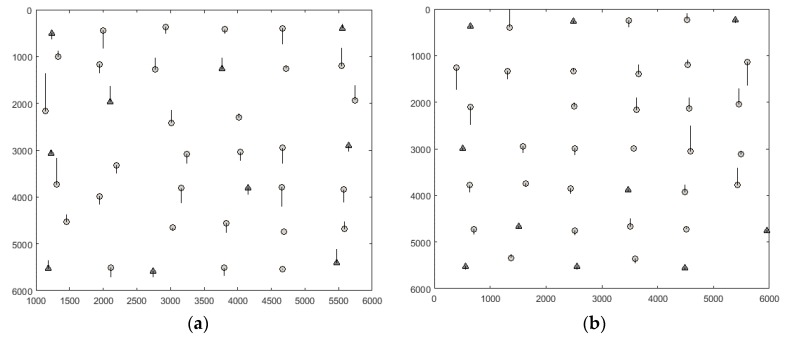

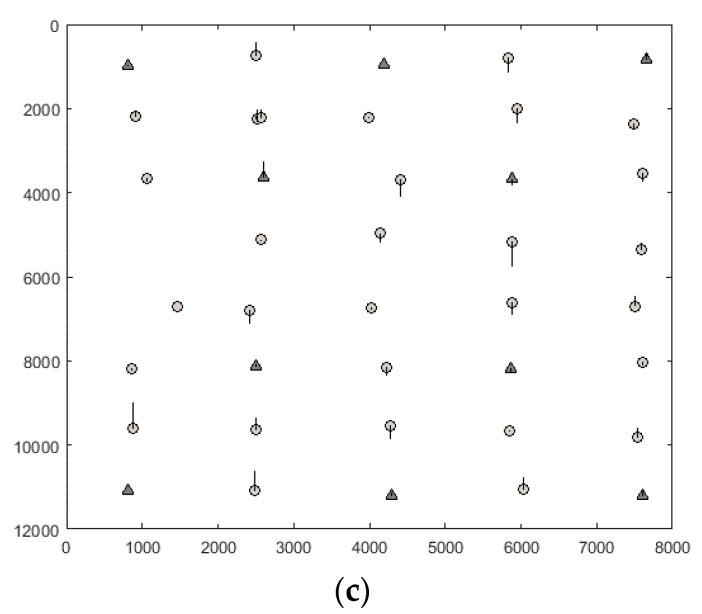
The scatter plot of the vertical parallaxes on the normalized imagery in Experiments 2, 6, and 10 with a magnification factor of 10,000: (**a**) Isfahan dataset; (**b**) Zanjan dataset; (**c**) Fars dataset.

Moreover, in order to examine the pattern of the vertical parallaxes on the normalized scenes, the scatter plot of the vertical parallaxes of CIPs in Experiments 2, 6, and 10 are shown in Figure 9. In this figure, the CoCP and ChCP are illustrated with the triangle and circle markers, respectively. The vertical parallaxes of CIPs are drawn with a magnification factor of 10,000.

According to Figure 9, a scatter plot of the residual vertical parallax of ChCP shows no systematic behavior. The proposed method has a low sensitivity to the number of CoCP used, as by increasing this number from 8 to 16, no appreciable variation in the results is observed. Generally, the minimum number of required CoCP can be variable regarding to the number of parameters used in the polynomials of Equations (12), (16) and (22). However, the distribution of these points on the stereo imagery is of great importance. Repetitive experiments show that selecting the CoCP around the outer boundary of the stereo images’ common coverage plays a key role on the accuracy of the model’s parameters (Figure 9).

As the final note on Table 4, the proposed model has a high ability to provide a linear relationship between the vertical parallax of ChCP and their heights in the object space. Since the height of ChCP have been calculated using the space intersection process and the EOP of original stereo imagery have been directly estimated using GCPs, the computed heights’ accuracy of ChCP depends on the accuracy and distribution of GCPs, along with the image coordinates’ accuracy of both the CoCP and ChCP.

**Figure 10:**
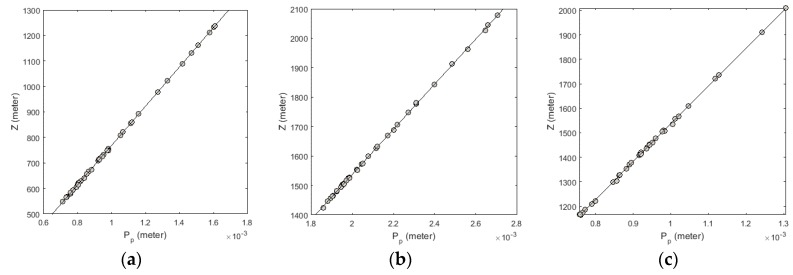
The scatter plot of the horizontal parallax of ChCP and their heights in Experiments 2, 6, and 10: (**a**) Isfahan dataset; (**b**) Zanjan dataset; (**c**) Fars dataset.

The scatter plots of the horizontal parallax of ChCP and their heights in Experiments 2, 6, and 10 are illustrated in Figure 10. According to Figure 10, the proposed method benefits from an admissible ability to provide a linear relationship between the vertical parallax of ChCP and their heights in the object space.

## 7. Conclusions

This paper presents a novel epipolar resampling approach for cross-track linear pushbroom images. The resampling process is based on the rigorous sensor model, with no approximations or assumptions. In this way, the epipolar curves of linear imagery are generally hyperbola-shaped, but if the scanlines containing the CIPs are parallelized with their IB, these curves are converted into straight lines. However, due to the temporal and spatial variation of the IB’s direction of CIPs, these epipolar lines will not be parallel to each other. Thus, in order to perform epipolar resampling of linear imagery by means of the rigorous sensor model, the attitude parameters of the stereo images should be modified and a complementary rotation should then be applied. Since the new attitude parameters and the complementary rotation angle are dependent to the IB’s direction of CIPs, using some polynomials in both the rows- and the columns-number of CIPs has been proposed for modeling these parameters. As the instantaneous position of sensors remains fixed, the DEM of the area of interest is not required in the resampling process. Given the EOP of stereo imagery, there is no need to any GCP, and the proposed method can be performed using only a few CIPs. Experimental results obtained from two pairs of SPOT-1A and one pair of RapidEye stereo imagery with different terrain conditions proved the feasibility and the success of the proposed method. One of disadvantages of the model is the need for geometrically raw stereo images, which isn’t offered by some providers. However, in the case of archive imagery and those which aren’t supplied with reliable RPCs, the proposed method can be very effective and almost the only option currently available.

Future studies will focus on a study of the feasibility of eliminating the need for CIPs to carry out the normalization procedure. Moreover, the proposed method will be developed for along-track stereo imaging systems, as well as those images captured by the manned and unmanned aerial platforms. Finally, DEM and orthophoto generation based on the normalized scenes will be considered.

## Figures and Tables

**Table 1:** Statistics of the polynomials fitted to the attitude parameters of the IB of CIPs.

Attitude Parameters	ω			ϕ			K		
Statistics	Value	*p*-Value	Std-Err	Value	*p*-Value	Std-Err	Value	*p*-Value	Std-Err
**β_0_**	0.449	4.4 × 10^−201^	1.0 × 10^−7^	0.140	1.5 × 10^−105^	2.1 × 10^−5^	0.061	2.4 × 10^−105^	9.3 × 10^−6^
**β_1_**	−1.6 × 10^−7^	9.3 × 10^−99^	3.9 × 10^−11^	−4.8 × 10^−10^	9.5 × 10^−4^	7.9 × 10^−9^	−2.1 × 10^−8^	7.7 × 10^−7^	3.5 × 10^−9^
**β_2_**	1.2 × 10^−8^	1.6 × 10^−55^	5.5 × 10^−11^	2.6 × 10^−6^	2.6 × 10^−56^	1.1 × 10^−8^	1.1 × 10^−6^	2.7 × 10^−56^	4.9 × 10^−9^
**β_3_**	3.6 × 10^−14^	1.6 × 10^−7^	5.6 × 10^−15^	−1.0 × 10^−11^	3.8 × 10^−11^	1.1 × 10^−12^	−5.1 × 10^−12^	5.0 × 10^−12^	5.0 × 10^−13^
**β_4_**	6.0 × 10^−13^	1.3 × 10^−44^	5.6 × 10^−15^	4.4 × 10^−12^	4.6 × 10^−5^	1.1 × 10^−12^	2.0 × 10^−12^	2.9 × 10^−5^	5.0 × 10^−13^
**β_5_**	1.0 × 10^−13^	3.4 × 10^−15^	7.5 × 10^−15^	1.8 × 10^−11^	5.3 × 10^−14^	1.5 × 10^−12^	8.6 × 10^−12^	1.2 × 10^−14^	6.7 × 10^−13^
**R^2^**	1.00			1.00			1.00		
${\overset{⌢}{\sigma }}_{0}$ **(s)**	9.0 × 10^−8^			1.8 × 10^−6^			8.1 × 10^−7^		
**Functional form:** Y = β_0_ + β_1_ × i + β_2_ × l + β_3_ × i × l + β_4_ × i^2^ + β_5_ × l^2^									

**Table 2:** Specifications of the datasets used.

Dataset	Isfahan		Zanjan		Fars	
**Platform**	SPOT-1		SPOT-3		Rapid Eye-2	
**Sensor**	HRV		HRV		Green Band ^†^	
**Acquisition date**	August 1987	January 1987	July 1993	July 1993	March 2010	March 2010
**Pointing angle**	24.7° W	20.84° E	19.01° W	16.66° E	19.64° W	7.09° E
**Ground resolution**	10 m		10 m		6.5 m	
**Base to height ratio**	0.974		0.737		0.534	
**Elevation relief**	687.2 m		654.2 m		842.6 m	
**Cloud percentage**	0%		>5%		>1%	

^†^ Bands of Rapid Eye’s Raw image data are not co-registered.

**Table 3:** Accuracy assessment of space resection and intersection of the stereo imagery.

Dataset		# Ctrls	# Chks	Space Resection (pix)			Space Intersection (m)			
				δr	δc	δrc	δX	δY	δXY	δZ
Isfahan	Scene 1	15	20	0.65	0.49	0.81	6.11	6.58	8.97	6.73
	Scene 2	15	20	0.76	0.54	0.93				
Zanjan	Scene 1	15	16	0.73	0.62	0.95	5.66	6.12	8.33	5.57
	Scene 2	15	16	0.56	0.7	0.89				
Fars	Scene 1	15	19	0.52	0.4	0.65	3.31	4.37	5.48	4.21
	Scene 2	15	19	0.55	0.38	0.67				

**Table 4:** Experimental results obtained from the proposed epipolar resampling method.

Dataset	Isfahan				Zanjan				Fars			
Experiment Number	1	2	3	4	5	6	7	8	9	10	11	12
Number of CoCP	8	10	12	16	8	10	12	16	8	10	12	16
Number of ChCP	32	30	28	24	32	30	28	24	32	30	28	24
Mean |P_V_|, pixels	0.03	0.02	0.00	0.00	0.02	0.01	0.01	0.00	0.03	0.02	0.02	0.01
Max |P_V_|, pixels	0.17	0.09	0.00	0.00	0.06	0.04	0.03	0.02	0.08	0.06	0.07	0.05
${\hat{\sigma }}_{0}$ (line fitting of P_H_ and Z), m	5.72	1.93	1.47	0.76	3.55	2.15	1.46	1.31	6.31	5.44	4.97	3.83

## Abbreviations

The following abbreviations are used in this manuscript:

IB	Instantaneous Baseline
CIPs	Conjugate Image Points
EG	Epipolar Geometry
DEM	Digital Elevation Model
ROP	Relative Orientation Parameters
EOP	Exterior Orientation Parameters
IOP	Interior Orientation Parameters
RFM	Rational Function Model
ACEL	Approximate Conjugate Epipolar Lines
RPCs	Rational Function Coefficients
MPC	Multiple projection Centers
PP	Parallel Projection
CCDs	Charged Coupled Devices
CMOS	Complementary Metal-Oxide Semiconductor
GCPs	Ground Control Points
IRF	Image Reference Frame
GRF	Ground Reference Frame
SRF	Sensor Reference Frame
PRF	Platform Reference Frame
CEL	Conjugate Epipolar Lines
CoCP	Control Conjugate Points
ChCP	Check Conjugate Points
